# Wiring a Periscope – Ocelli, Retinula Axons, Visual Neuropils and the Ancestrality of Sea Spiders

**DOI:** 10.1371/journal.pone.0030474

**Published:** 2012-01-18

**Authors:** Tobias Lehmann, Martin Heß, Roland R. Melzer

**Affiliations:** 1 Bavarian State Collection of Zoology, Munich, Germany; 2 Department Biology I, Ludwig-Maximilians-Universität München, Planegg-Martinsried, Germany; Lund University, Sweden

## Abstract

The Pycnogonida or sea spiders are cryptic, eight-legged arthropods with four median ocelli in a ‘periscope’ or eye tubercle. In older attempts at reconstructing phylogeny they were Arthropoda incertae sedis, but recent molecular trees placed them as the sister group either to all other euchelicerates or even to all euarthropods. Thus, pycnogonids are among the oldest extant arthropods and hold a key position for the understanding of arthropod evolution. This has stimulated studies of new sets of characters conductive to cladistic analyses, e.g. of the chelifores and of the hox gene expression pattern. In contrast knowledge of the architecture of the visual system is cursory. A few studies have analysed the ocelli and the uncommon “pseudoinverted” retinula cells. Moreover, analyses of visual neuropils are still at the stage of Hanström's early comprehensive works. We have therefore used various techniques to analyse the visual fibre pathways and the structure of their interrelated neuropils in several species. We found that pycnogonid ocelli are innervated to first and second visual neuropils in close vicinity to an unpaired midline neuropil, i.e. possibly the arcuate body, in a way very similar to ancestral euarthropods like *Euperipatoides rowelli* (Onychophora) and *Limulus polyphemus* (Xiphosura). This supports the ancestrality of pycnogonids and sheds light on what eyes in the pycnogonid ground plan might have ‘looked’ like. Recently it was suggested that arthropod eyes originated from simple ocelli similar to larval eyes. Hence, pycnogonid eyes would be one of the early offshoots among the wealth of more sophisticated arthropod eyes.

## Introduction

Sets of neuroanatomical characters have contributed important arguments to the discussion about the phylogenetic position of Pycnogonida. Lately, studies of the first head segment [1] in Pycnogonida and of its appendages, the chelifores [2],[3],[4] have shown that the innervation of the protocerebrum is promising in this respect. In arthropods the protocerebrum's sensory parts are primarily responsible for the visual system. Due to its phylogenetic relevance the latter is well studied [5], as exemplified by the Tetraconata concept (Crustacea + Insecta), in which the structure of the eyes is eponymous [6],[7]. In many arthropods both lateral and median eyes occur, pycnogonids possess only a periscope-like ocular tubercle with four ocelli generally interpreted as median eyes, whereas classical lateral eyes are absent. The visual system of sea spiders is sparsely examined, which is surprising considering their key role as basal chelicerates/arthropods. The eyes of littoral species – which are also used for this study – exhibit an optimum light sensitivity of between 530–545 nm, similar to many marine invertebrates which occupy a comparable habitat [8]. Probably their most important function is to orientate the animal to the incident light [8]. The quadruple of median ocelli in sea spiders seem to represent an ancestral character state of median eyes in Arthropoda and/or Euarthropoda [5], and correspond well to what might be precursors of nauplius eyes and median eyes in other arthropods. Remarkably, only few taxa have been studied in detail with light [9],[10] and electron microscope [11],[12], revealing some features typical of median eyes, i.e. that they are pigment cup ocelli with latticed rhabdom, surrounding pigment layers, and cuticular lens. Conversely, the structure of the retinula or R-cells that could be described as “pseudoinverted”, and the presence of a tapetum lucidum (guanine multilayer reflector) might be derived conditions. This very uncommon retinula cell architecture shows more similarities to the lateral eyes of spiders than to ‘normal’ median eyes [12]. Notably, our knowledge of the visual neuropils connected to the eyes is also cursory at this time. Hanström's [13] classical study suggested some putative visual neuropils and their fibre connections based on classical histology (with a few addenda contributed by Winter [14]), but they have never been identified using unequivocal markers or tracers. Deeper knowledge of, e.g., R-cell projections and visual neuropil architecture is missing, hence there is no stable basis on which to compare visual system features among pycnogonids and to those of their putative arthropod outgroups. In Chelicerata other than Pycnogonida, the visual systems of *Limulus polyphemus* [15],[16],[17] and *Cupiennius salei* [18],[19], which are important model organisms in the field of visual neuroscience, are especially well studied. In scorpions the only study of the visual neuropils is that of Hanström [13].

In the present study we therefore use a multiple-method (3D semithin serial reconstruction, transmission EM, Wigglesworth stains, cobalt backfills, Golgi technique) and multiple-species (*Achelia langi*, *A. vulgaris*, *Endeis spinosa*) approach. The visual neuropils are identified, and their basic architecture is analysed along with the termination sites of retinula cell axons, revealing basic features of the visual system generally studied in Arthropoda to allow comparison with other arthropod lineages.

## Results

**Figure 1:**
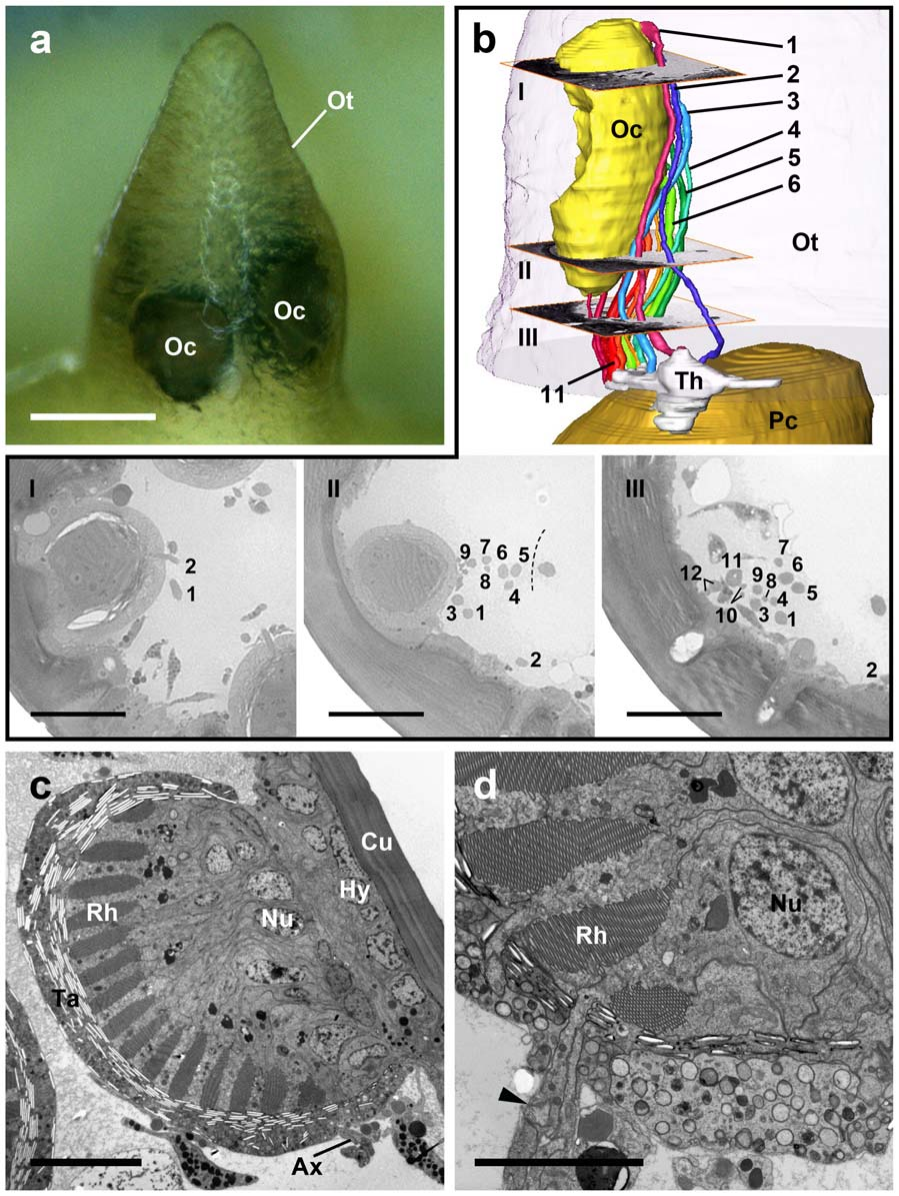
Periscope-like ocular tubercle with ocelli and nerve fibres to the protocerebrum. **a**, Light microscopic picture of the ocular tubercle (Ot) in *Endeis spinosa* showing two of the four ocelli (Oc). Bar 100 µm. **b**, 3D semithin serial reconstruction of nerve fibres projecting from left rostral ocellus to dorsolateral thickening distal to first neuropil (*Endeis spinosa*). Note retained relative positions of nerve fibres representing subsets of retinula cells (indicated by numbers). I–III: Three selected planes (Richardson staining; for position, see rendering at top right), showing profiles of groups of photoreceptor nerve fibres, originating from neighbouring r-cells, indicated by numbers. I, Frontal section from top quarter of eye. II, Frontal section from bottom quarter of eye. III, Frontal section through loose strand of nerve fibres just below eye. Bars 25 µm. Oc, ocelli; Ot, ocular tubercle; Pc, protocerebrum; Th, thickening. **c**, Transmission EM of a single ocellus in *Achelia vulgaris* showing the arrangement of the retinula cells. Ax, axon; Cu, cuticle; Hy, hypodermis; Nu, nucleus; Rh, rhabdom; Ta, tapetum. Bar 5 µm. **d**, Transmission EM of a retinula cell with a sequence from outside to inside of nucleus (Nu), rhabdom (Rh), and axon (arrowhead) demonstrating their “pseudoinverted” structure (*Achelia vulgaris*). Bar 10 µm.

**Figure 2:**
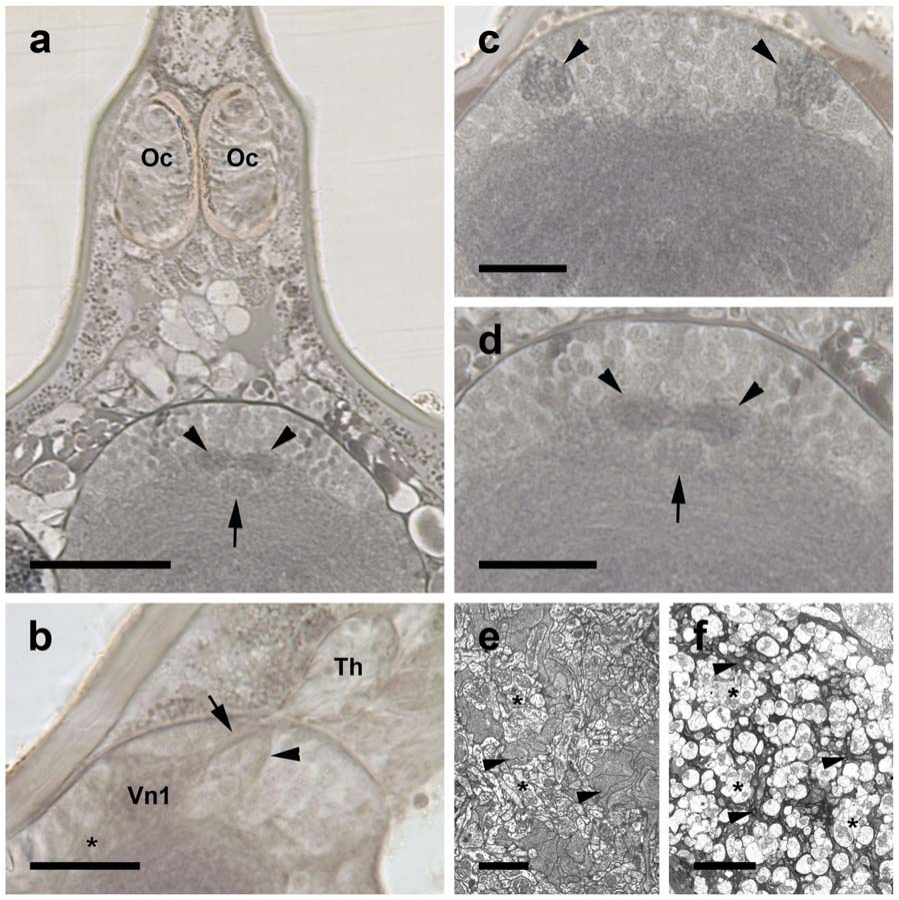
Anatomy of the visual neuropils (a–d, Wigglesworth stains, *Achelia vulgaris*; e, f TEM, *Endeis spinosa*). Note dark stain of sensory neuropils after application of Wigglesworth's technique. **a**, Eye tubercle with two ocelli (Oc) and protocerebrum with left and right second visual neuropils (arrowheads) and arcuate body (arrow), transversal section. Bar 50 µm. **b**, Thickening (Th) distal to protocerebral cell body rind, first visual neuropil (Vn1), bifurcation of visual tract into a subset of fibres projecting to first (arrow) and second neuropil (arrowhead), respectively, and tract connecting first visual neuropil with protocerebrum (asterisk), sagittal section. Bar 25 µm. **c**, First visual neuropils (arrowheads) dorsolaterally in rostral part of protocerebrum, transversal section. Bar 25 µm. **d**, Second visual neuropils (arrowheads) deeper in protocerebrum in a more rostral and central position, and arcuate body (arrow), transversal section. Bar 25 µm. **e**, **f**, Frontal section of distal (**e**) and (**f**) of proximal region of first visual neuropil showing arrangement of retinula axon terminals (arrowheads) and dendrites and cell bodies of visual second order neurons (asterisks). Note that in distal region (**e**) retinula axons are broad; in proximal region (**f**) they are narrow. Bars 5 µm.

**Figure 3:**
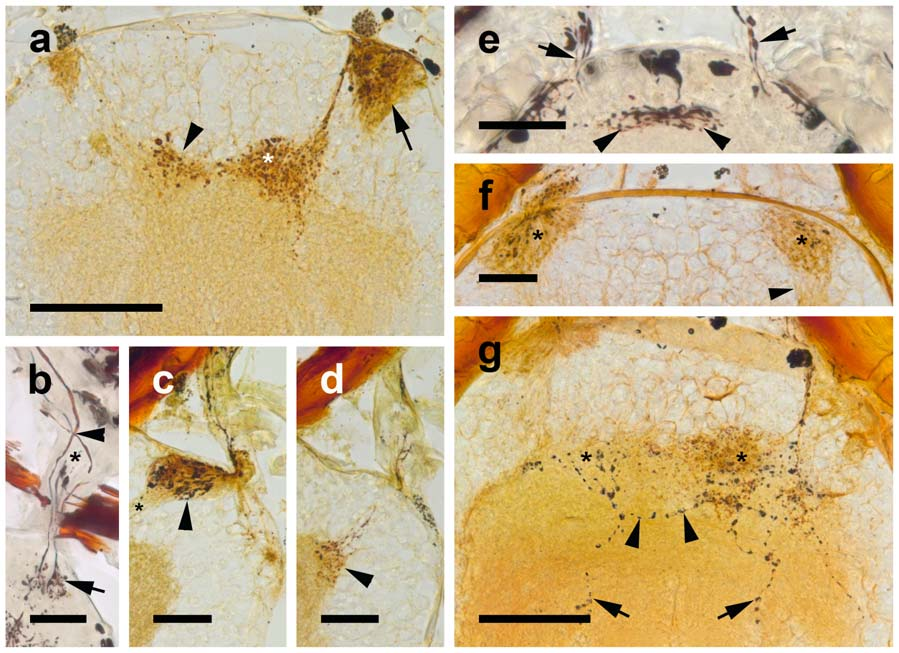
Neuroanatomy of the visual neuropils revealed with cobalt backfills (a, c, d, f, g) and Golgi technique (b, e). **a**, **b**, *Achelia langi*; **c**–**e**, *Achelia vulgaris*; **f**, **g**, *Endeis spinosa*. In **a** and **g** cobalt backfills of two sections are combined. **a**, First (arrow) and second (asterisk) visual neuropil identified with cobalt backfills, transversal section. Note dense arrangement of cobalt filled profiles in both neuropil pairs. Arrowhead points to a few axons of the right retinula cells that send axon collaterals to the contralateral, left neuropil. Bar 50 µm. **b**, Retinula axons projecting from dorsal through dorsolateral thickening (asterisk) into first visual neuropil (arrow) where they form short collaterals and synaptic varicosities; note re-assortment of single axons (arrowhead), transversal section. Bar 25 µm. **c**, **d**, Retinula axon terminals in first (**c**) and second (**d**) visual neuropil, with synaptic varicosities in both neuropils (arrowheads); in c a tract connects first visual neuropil with protocerebrum (asterisk); sagittal sections. Bars 25 µm. **e**, Retinula axons (arrows) and second visual neuropils (arrowheads), transversal section. Bar 25 µm. **f**, **g**, Cobalt backfills of retinula axons terminating in first (**e**) and second (**f**) visual neuropils (asterisks); in **f** a tract connects first visual neuropil with protocerebrum (arrowhead); in **g** a fibre connecting ipsi- and contralateral second neuropil is seen (arrowheads), note a single fibre per brain hemisphere that travels through second visual neuropil and terminates deeper in protocerebrum (arrows); transversal sections. Bar 50 µm.

The visual system of the studied pycnogonids is composed of (from distal to proximal): four ocelli in a periscope-like eye tubercle (Fig. 1a); several nerve fibres projecting from the eyes proximal to the dorsal protocerebrum (Fig. 1b); a dorsolateral thickening where the nerve fibres from the two eyes of one hemisphere concentrate without forming synaptic varicosities before entering the protocerebrum (Fig. 1b, 2b, 3b); and two successive distinct visual neuropils prepossessed by R-cell axons and terminals in each brain hemisphere where the retinula cells terminate (Fig. 3). Furthermore, there is an unpaired midline neuropil in the central protocerebrum located underneath the second visual neuropils.

Transmission EM of *Achelia vulgaris* confirms the “pseudoinverted” structure of the ocelli also for this species (Figs. 1c, d). Each of the four ocelli is connected to the brain via several nerve fibres originating in a consorted manner in the form of a dorsoventral row from the inner side of the ocelli (Fig. 1b). These fibres are composed of a few axons from neighbouring retinula cells and hence represent one part of the retina, i.e. one sector of the visual environment. The fibres join successively, and finally the nerves of the two left and accordingly the two right ocelli combine in a thickening dorsolaterally on each brain hemisphere just before they enter the protocerebrum (Figs. 1b, 2b, 3b–d). The 3D-reconstruction of *Endeis spinosa* shows a primitive form of retinotopic projection arrangement of these nerve fibres, since they maintain the same order – from top to bottom of each ocellus – as they enter the thickening before the brain (Fig. 1b), i.e. nerve fibres originating from the dorsalmost eye portion enter the thickening caudally, and the ventralmost fibre projects to its rostral part. In this thickening all nerve fibres from the eyes are bundled and a re-assortment of the single axons takes place (Fig. 3b), but a typical neuropil architecture caused by fine dendritic arborisations and axon collaterals was not detected. Cobalt backfills via the ocelli in *Achelia langi*, *A. vulgaris* and *Endeis spinosa* reveal two distinct retinula axon target regions in each hemisphere of the protocerebrum, a first and a second visual neuropil (Fig. 3). The first neuropil is located dorsolaterally in the rostral part of the protocerebrum as an oval-shaped region laterally embedded in the cell body rind of the brain (Fig. 2b, c). The second neuropil lies deeper, under the cell body rind and in a more rostral and central position in the protocerebrum. The second neuropils of both brain hemispheres contact each other in the brain's midline, and are dumbbell-shaped when seen together (Fig. 2a, d).

After entering the brain the fibre bundle is split; one part of the axons has its terminals in the first visual neuropil (Fig. 3a, b, c, f), the other part passes the first one and terminates in the second neuropil (Fig. 3a, d, e, g). This division is also observed by TEM and Wigglesworth stains in *Achelia vulgaris* (Fig. 2b). With the latter method, the first and second visual neuropil can be recognised as dark-stained areas, as is typical for Wigglesworth-stained sensory neuropils (Fig. 2). In addition, a tract originating from the first neuropil has been identified that projects basally into the protocerebrum (Figs. 2b; 3c, f). Axially beneath the left and right second visual neuropil lies a roundish, unpaired midline neuropil, also somewhat darker-stained, which can be identified as the arcuate body (Fig. 2a, d).

Transmission EM of the first visual neuropil reveals several clusters of cells with high electron density, identified as retinula axon terminals, surrounded by cells with low electron density, identified as second order neurons, with synapses between these neurons (Fig. 2e, f). In the distal region of the visual neuropil these cells fill a large part of the neuropil, in the proximal region they taper off (Fig. 2e, f). At least some of the second order neurons likely project deeper into the protocerebrum – via the tract shown in Figures 2b and 3c, f – hence are visual interneurons.

Golgi-impregnated brains of *Achelia langi* and *A. vulgaris* show that in this neuropil the terminals are branched and have synaptic varicosities (Fig. 3b). In the second visual neuropil, cobalt backfills identify only varicosities with certainty, whereas branching is suggested only (Fig. 3a, d, e, g). Axons of the right and left second visual neuropils contact each other medially (Fig. 3e, g); a few axons of the right retinula cells also terminate in the contralateral left neuropil, and vice versa (Fig. 3a). This is supported by cobalt backfills in which retinula cells of only one hemisphere are stained, but terminals that also end in the contralateral neuropil can be identified. Furthermore, one single axon per brain hemisphere travels through the second visual neuropil and terminates even deeper in the brain (Fig. 3g), with varicosities all over its extension.

## Discussion

Our studies confirm that the brain area described by Hanström [13] as “*Sehmasse*” is a genuine visual neuropil. This neuropil was also found by Winter [14] (“*Seemasse 1*”). In addition he suggested the presence of a second visual neuropil (“*Seemasse 2*”) postero-ventrally adjacent to the first neuropil, but this one was not stained by our cobalt backfills, though a tract projecting to this region is identifiable in our stains. If present, this neuropil would therefore not be a target of visual fibres, but of visual interneurons.

Conversely, the brain area interpreted by Winter [14] as the calyx of the mushroom body corresponds in position and shape exactly to the second visual neuropil that we identified with cobalt backfills. How can this contradictory result be explained? Winter described the mushroom body without going into detail; his observations were based on classical histology only. He named a region under the cell body rind as paired “Corpora pedunculata”, which equates to the calyx of mushroom bodies [20], with ventrally adjacent “Stielelementen”, which equate to the pedunculus of mushroom bodies [20]. In the meantime Strausfeld *et al.* [21] described a different brain area as the mushroom body. In this interpretation the mushroom body lobes were characterised – like those of onychophorans – as horseshoe-shaped, and as confluent across the midline of the protocerebrum, but a primitive nature was suggested. This indicates that Winter might have misinterpreted the mushroom body. This view is also supported by the present findings, since the mushroom bodies in arthropods are generally not innervated by median eye retinula axons [21], and the neuropil in question is unequivocally identified here as a visual neuropil.

Furthermore, we possibly localised the arcuate body in a position of the protocerebrum different from the one suggested by Hanström or Winter (“*Zentralkörper*” [13],[14]), i.e. right beneath the second visual neuropils, a region not specified by those authors. In the chelicerates only one unpaired midline neuropil in the protocerebrum is known, the arcuate body [22]. It has a dorso-posterior position in the brain's midline and is closely related to the visual system [22],[23]. The same features are found here for pycnogonids, although this neuropil is not as complex as in other chelicerates or onychophorans but rather small. Thus, this neuropil may be the arcuate body of pycnogonids, but more research about this issue will have to be done.

Thus, our study leads to a new interpretation of the visual system as well as of the general architecture of the pycnogonid protocerebrum. The visual system comprises three main elements: (1) a thickening where the retinotopic nerve fibres from the median eyes are docking and re-assorted; (2) a first and (3) a second visual neuropil, each targeted by subsets of the retinula axon terminals; and (4) the second visual neuropil is located in close vicinity to an unpaired midline neuropil, possibly the arcuate body. Furthermore, there are projections to the contralateral second neuropil and fibres projecting to centres located deeper in the protocerebrum. These highly specific features allow a detailed comparison with the situation found in other arthropods.

In Tetraconata or Pancrustacea one finds only a single median ocellar nerve with terminals of the ocellar photoreceptor in the dorso-median protocerebrum (e.g. *Balanus nubilus* [24] and *Schistocerca gregaria* [25]). In Myriapoda median eyes are absent. In Chelicerata and Onychophora the projections of the median eye nerves differ fundamentally from those in Mandibulata – and are similar to the pycnogonid condition found here – in having a paired nerve that connects the eyes with the brain. In derived taxa such as the spider *Cupiennius* [18] there is only one target region of the retinula axon terminals of the median eyes (principal eyes or anterior median eyes): the first anterior median eye neuropil, dorso-lateral in each brain hemisphere. A similar situation is found in scorpions [13], and it differs from our findings on pycnogonids. Conversely, in *Limulus* [16] two target regions of the median eyes in each brain hemisphere exist: the two ocelli are indeed only innervated to the ocellar ganglion, but the fused rudimentary median eye is innervated to the ocellar ganglion and simultaneously to a region near the central body, as also shown here for sea spiders. In Onychophora (*Euperipatoides rowelli*) [26],[27], one of the putative sister taxa of Euarthropoda, the presence of photoreceptor terminals in a first visual neuropil, which lies directly beneath the eye, was suggested [26],[27]. From this first neuropil, an optic tract projects further and then bifurcates as in pycnogonids. Its ventral branch extends to a second visual neuropil near the mushroom body calyces, while the dorsal branch gives rise to another second visual neuropil, which flanks the arcuate body laterally. Thus, comparing the median eye visual system of pycnogonids to that of other (pan)arthropods, the similarities are greatest to xiphosurans and onychophorans, intermediate to spiders and scorpions, and lowest to mandibulate arthropods.

The dorso-posterior position of the pycnogonid arcuate body is also in accordance with that in other chelicerates and in onychophorans (see review by Homberg [22]), but in *Limulus* and arachnids it is more or less horseshoe-shaped, and in Onychophora it is subdivided in lamina posterior and lamina anterior. In these taxa the arcuate body is associated with the visual system, in *Limulus* and arachnids actually with the median eyes [22]. The close vicinity to the second visual neuropils leads one to assume that in pycnogonids the arcuate body is also associated with the visual system.

The similarities between Pycnogonida and Onychophora and Xiphosura, the two taxa with the greatest accordance, are that all three taxa have (1) a paired nerve that connects the eyes with the brain; (2) two visual neuropils within the brain connected to (median) eyes; and (3) that one of the visual neuropils lies in direct vicinity to an unpaired midline neuropil, i.e. arcuate body. But there are also differences to these two taxa; in *Limulus* only the axons of the fused rudimentary median eye has these two target regions (the axons of the two other median eyes all end in the ocellar ganglion), and these retinula axons have some branches both in the ocellar ganglion and in the region near the central body. In pycnogonids the retinula axons have branches only in the first or second visual neuropil, and never in both neuropils simultaneously. In onychophorans there are three visual neuropils: one first visual neuropil beneath the eye, and two second neuropils within the brain; in pycnogonids only two genuine neuropils containing R-cell axon terminals and the distal thickening are found. However, bifurcation of visual tracts is found only in Onychophora and Pycnogonida. In onychophorans it has not been analysed whether the photoreceptor axons terminate in the first visual neuropil only or also in the second neuropils. This would be valuable information for further comparisons.

Features that might be unique to sea spiders, as they have not been found in other arthropods, are that some of the terminals of retinula axons end in the contralateral second visual neuropil, and that fibres project to deeper areas of the protocerebrum.

**Figure 4:**
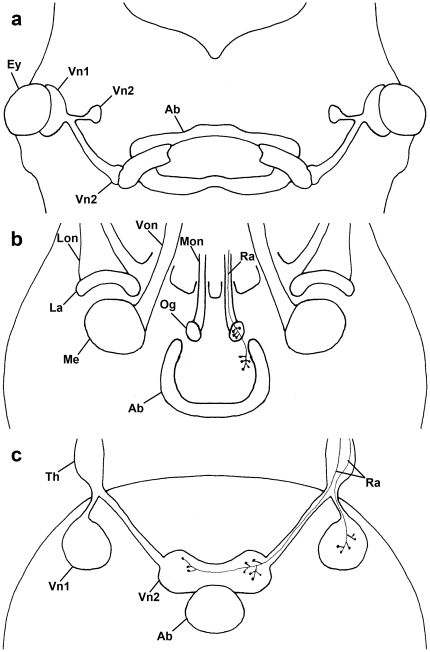
Comparison of visual systems of (a) Onychophora (*Euperipatoides rowelli*), (b) Xiphosura (*Limulus polyphemus*), and (c) Pycnogonida (*Achelia* spp., *Endeis spinosa*). Ab, arcuate body; Ey, eye; La, lamina; Lon, lateral optic nerve; Me, medulla; Mon, median optic nerve; Og, ocellar ganglion; Ra, retinula axon; Th, thickening; Von, ventral optic nerve; Vn, visual neuropil. **a**, Visual pathways from the eyes are shown, with first and second optic neuropils indicated. After Strausfeld *et al.* [*27*]. **b**, Terminals of median rudimentary photoreceptor have some branches in ocellar ganglion, then continue and terminate near central body. After Calman *et al.* [16]. **c**, Summary of the situation found in the three pycnogonid species studied here.

The sets of characters studied here for pycnogonids and those of other arthropods are summarised in the data matrix given in Table 1 and in Figure 4. The visual system in sea spiders shows far more similarities to those in basal xiphosurans and even in an arthropod outgroup – oynchophorans – than to those in derived chelicerates like scorpions and spiders (Table 1, Fig. 4). This represents another argument for placement of the sea spiders at the base of the Chelicerata or even Euarthropoda, as suggested by recent molecular trees [28], [29].

**Table 1:** Data matrix with pycnogonid eye features (this study) compared to median eyes of other arthropods (citations for exemplary taxa used are given above).

Feature	Onychophora *Euperipatoides rowelli*	Pycnogonida *Achelia spp., Endeis spinosa*	Xiphosura* *Limulus polyphemus*	Arachnida *Cupiennius salei*	Crustacea *Balanus nubilus*	Hexapoda *Schistocerca gregaria*
A	0	0	0	0	1	1
B	0	0	0	0	1	1
C	-	0	0	1	1	1
D	0	0	1	1	1	1
E	-	0	0	1	1	1

A, eye nerves paired and arranged in bilateral symmetry (0) or unpaired (1); B, visual neuropils paired and arranged in bilateral symmetry (0) or unpaired ocellar centre (1); C, number of visual neuropils innervated by R-cell axons greater than one (0) or equal to one (1); D, bifurcation of subsets of visual fibres targeting two different neuropils present (0) or absent (1); E, second visual neuropil with visual fibre terminals in close vicinity to arcuate body present (0) or absent (1). Due to absence of median eyes, Myriapoda are omitted; “-“ indicates that the feature has not been studied.

*characters of fused rudimentary median eye.

The fact that the visual system of pycnogonids shows more similarities to the fused rudimentary median eye of *Limulus* than to the ‘normal’ median eye, is of special interest. If arthropod eyes originated from simple ocelli similar to larval eyes [30], pycnogonid eyes could be one of their early offshoots, which date back at least 500 Myr to the Cambrian [31], and be older than the appearance of distinct lateral and median eyes.

## Materials and Methods

### Specimen collection

The specimens of *Achelia langi, A. vulgaris* and *Endeis spinosa* were collected during field trips in 2009 and 2010 to Rovinj (Croatia), Isola del Giglio (Italy), and Roscoff (France).

### 3D-Reconstruction

Eye tubercle (prepared as for TEM) was cut into a most complete semithin cross-section series (1 µm) using a HistoJumbo diamond knife on a RMC-MTXL ultramicrotome. The slices were mounted on glass slides, stained with methylene blue (after Richardson *et al.* [32]), coverslipped and photographed with a conventional light microscope (40x, NA 0.95). The images were contrast enhanced in Photoshop and then aligned, segmented and rendered in Amira.

### TEM

After dissection of abdomen, legs and proboscis the animals were fixed in 4% glutardialdehyde in 0.1 M cacodylate buffer at 4°C, postosmicated and embedded in epoxy resin. Ultra-thin sections of 70–100 nm thickness were made with a diamond knife on an RMC-MTXL ultramicrotome. The sections were stained with uranyl actetate and lead citrate, and inspected in an FEI Morgagni transmission EM at 80 kV.

### Osmium-Ethyl Gallate procedure (modified after Wigglesworth [33])

After dissection of abdomen, legs and proboscis the animals were fixed in 3% glutardialdehyde in 0.1 M cacodylate buffer at 4 °C. After postosmication the animals were stained for 48 hours at 4 °C in a saturated ethyl gallate solution, dehydrated, kept overnight in methyl benzoate, embedded and sectioned (5–8 µm).

### Cobalt backfills (modified after Altman & Tyrer [34])

CoCl_2_ crystals were inserted in one or two ocelli with a tungsten needle. After diffusion times between 1 and 5 hours, cobalt was precipitated with (NH_4_)_2_S solution. Animals were fixed in AAF (ethanol, glacial acetic acid, formaldehyde), silver intensified, embedded, and sectioned (10–12 µm).

### Golgi technique

Abdomen, legs and proboscis were dissected and the cuticle regions surrounding the central nervous system were perforated in order to increase the chances for staining the desired areas. The preparations were submitted to two cycles of the Golgi-Colonnier method [35], embedded and sectioned (10–20 µm).

### Terminology

All neuroanatomical terms and definitions were adopted from Richter *et al.* [20].
